# Assisting Phytoremediation of Heavy Metals Using Chemical Amendments

**DOI:** 10.3390/plants8090295

**Published:** 2019-08-21

**Authors:** Md. Mahadi Hasan, Md. Nashir Uddin, Iffat Ara-Sharmeen, Hesham F. Alharby, Yahya Alzahrani, Khalid Rehman Hakeem, Li Zhang

**Affiliations:** 1 State Key Laboratory of Grassland Agro-Ecosystems, School of Life Sciences, Lanzhou University, Lanzhou 730000, China; 2 Department of Biochemistry and Microbiology, School of Health and Life Sciences, North South University, Dhaka 1229, Bangladesh; 3 Department of Biochemistry, School of Life Science, Independent University, Dhaka 1229, Bangladesh; 4 Department of Biological Sciences, Faculty of Science, King Abdulaziz University, Jeddah 21577, Saudi Arabia; 5 Institute of Cell Biology, School of Life Sciences, Lanzhou University, No.222 South TianShui Road, Lanzhou 730000, China

**Keywords:** environmental pollution, phytoextraction, cadmium, biostimulation, oxidative damage

## Abstract

Phytoremediation is one of the safer, economical, and environment-friendly techniques in which plants are used to recover polluted soils, particularly those containing toxic organic substances and heavy metals. However, it is considered as a slow form of remediation, as plants take time to grow and flourish. Various amendments, including the augmentation of certain chemical substances i.e., ethylenediamine tetraacetic acid (EDTA), ethylene glycol tetra acetic acid (EGTA), and sodium dodecyl sulfate (SDS) have been used to induce and enhance the phytoextraction capacity in plants. Several reports show that chemical amendments can improve the metal accumulation in different plant parts without actually affecting the growth of the plant. This raises a question about the amount and mechanisms of chemical amendments that may be needed for potentially good plant growth and metal phytoremediation. This review provides a detailed discussion on the mechanisms undertaken by three important chemical amendments that are widely used in enhancing phytoremediation (i.e., EDTA, EGTA, and SDS) to support plant growth as well as soil phytoremediation. A core part of this review focuses on the recent advances that have been made using chemical amendments in assisting metal phytoremediation.

## 1. Introduction

Heavy metal pollution is regarded as a serious problem for crop production [1]. In soil ecosystems, the gradual increase in heavy metal levels is a major concern throughout the world [2]. Heavy metal pollution is a vital issue for environmental management due to the rapid increase in anthropogenic activities, including industrialization, transportation, and urbanization. Several sources of heavy metals include medical waste, the combustion of coal, petrol, mining, fertilizers, smelting, and pesticides, which are adding heavy metals to the environment [3,4]. Among these heavy metals, cadmium (Cd), lead (Pb), aluminum (Al), zinc (Zn), manganese (Mn), chromium (Cr), and copper (Cu) are considered as common toxic heavy metals [1].

Toxic heavy metals considered as phytotoxic agents to plants that affect the plant morphological and physiological processes such as lower growth rate, stomatal movement and nutrient imbalance, and photosynthetic processes result in the oxidative damage [5]. Due to increased contamination of soil by toxic metal components, it is important to use necessary techniques for cleaning up heavy metals from contaminated soil, which requires an effective and reasonable solution. Some modern techniques that have been used for the remediation of heavy metals are based on the biological, chemical, and physical approaches [6]. Phytoremediation is one of the biological approaches used for the remediation of soils. Phytoremediation is preferable due to its safety and lower cost as compared to physical and chemical remediation [6]. The technologies of metal phytoremediation include phytoextraction, phytostabilization, and phytovolatilization [7]. Among these, phytoextraction is one of the promising techniques being used for reclaiming the metal polluted soils [1]. 

However, the heavy metal extraction by plants is usually limited by the availability of heavy metals in soils. The application of chemical enhanced technology is one of the prevalent approaches, which helps the uptake of heavy metals and their translocation in the aboveground parts of plants [4,8]. 

Several chemical amendments have been used for the enhancement of phytoextraction processes. In recent years, chemical amendments including ethylenediamine tetraacetic acid (EDTA), ethylene glycol tetraacetic acid (EGTA), and sodium dodecyl sulfate (SDS) are widely used for soil remediation applications, which can bring potential metal leaching risk. EDTA is considered as having the most potential and most studied chemical amendment, and is used to increase the phytoextraction of metals from contaminated soils [9].

Therefore, it is important to increase phytoremediation efficiency through the phytoextraction processes of heavy metals. The phytoremediation of heavy metals in plants may be enhanced through an emerging chemical amendments technology. Our present review attempts to describe the potential benefits of chemical amendments in phytoremediation research.

## 2. Source of Heavy Metal Pollution, Ecotoxicity, and Approaches for Remediation

**Figure 1:**
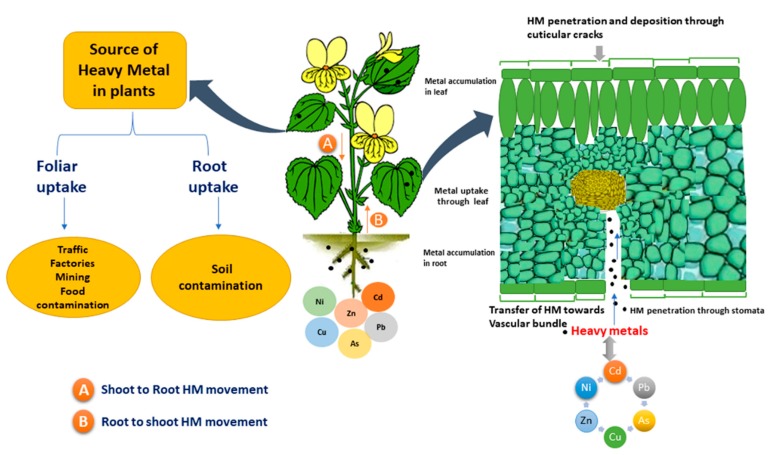
Sources of heavy metals, and foliar, root uptake of heavy metals in plants.

In general, heavy metals originate from anthropogenic and natural sources. Different sources of heavy metals were identified such as (a) industrial sources, (b) domestic effluent, (c) agricultural sources, and (d) natural sources such as the atmosphere (Figure 1). It has been reported that most of the areas in the world such as China, Japan, and Indonesia have been contaminated by Cd, Cu, and Zn due to mining and agricultural operations [6].

In terrestrial ecosystems, the heavy metal contaminations are increasing due to anthropogenic activities, which are known to be liable for ecotoxicity. The largest availability of heavy metals toxicity occurs in soil and aquatic ecosystems, whereas the smaller portion of metals available in the atmosphere form of vapors or particulate. In soil, the major heavy metal ranges vary from 0.01 to 0.7 ppm dw of Cd, 2 to 200 ppm dw of Pb, 10 to 300 ppm dw of Zn, 5 to 3000 ppm dw of Cr, and 7000 to 55,000 ppm dw of Fe etc. [10]. Heavy metal pollution in soils is becoming increasingly common in the agricultural sector, and affects food safety and crop growth. The metal toxicity in plants is not the same; it varies with plant species, metal concentration, soil pH, soil composition, specific metal and chemical form, etc. According to Misra and Mani [11], the range of vital heavy metal in plants is 0.1 to 2.4 µg^−1^ dw for Cd, 1 to 13 µg^−1^ dw for Pb, 0.02 to 7 µg^−1^ dw for As, 8 to 100 µg^−1^ dw for Zn, 0.2 to 1 µg^−1^ dw for Cr, 140 µg^−1^ dw for Fe, etc.

Toxic heavy metal is hazardous to the environment. Therefore, heavy metal remediation in the soil is very important [12]. Over the past few decades, various techniques were employed for the remediation of toxic heavy metals. The preferred strategies were selected for the remediation of heavy metal from the environment based on their technical complexity and cost [13]. The techniques include physical, chemical, and biological methods [14].

Traditionally, the contaminated soils are cleaned up by excavation or removing the soils from the land sites. The toxic metals problems in the soil may also arise due to the transportation of contaminants that are closely adjacent to the soils [15]. Soil washing is another strategy to eliminate soil metal contaminants, but it has been reported that this is unsuitable for plant growth and development due to the hindrance of biological and chemical activities [16]. Chemical methods are not preferable due to alterations in the soil texture and structure, expense, and the generation of high quantities of sludge [16,17].

### 2.1. Physiochemical Techniques

The physiochemical technique includes excavation, leaching, landfill, and thermal treatment (bioreactor) approaches. Nevertheless, these processes are fast but costly, and have detrimental effects on the soil properties [13,18]. These techniques are not completely suitable for heavy metal remediation, and only change the form of the problem without remediating the pollutants thoroughly [19].

### 2.2. Biological Approaches 

Heavy metals are removed from the environment through natural remediation coordinated by microorganisms and plants [14]. Biological remediation is a preferable method as it is natural, cost-effective, environmentally friendly, and has wider public support [20]. There are several approaches, such as bioaugmentation, biostimulation, bioleaching, composting, bioreactors, bioremediation, and phytoremediation [21]. Biological approaches are considered superior to physiological approaches, because these processes use solar energy and ensure that the soil properties are conserved [20]. Bioremediation is a technique by which heavy metals are removed from the environment [19,22]. Bacterial strains such as *P. aeruginosa* and *Bacillus* spp. can remediate metals such as zinc and copper in this context [23]. Bioremediation can be done using biostimulation, biofilters, treated and pumped methods, bioventing, bioreactors, composting, land forming, bioaugmentation, and intrinsic bioremediation [24]. The efficiency of phytoremediation can be increased if microbes are used [25]. 

#### 2.2.1. Phytoremediation of Heavy Metals

Phytoremediation is a part of the emerging green technology being used for the uptake of various heavy metals in different amounts from the soil and storage of them in parts of the plant that can be harvested [26]. With changes in soil properties, plants can tolerate pollutants [27]. Soils containing heavy metals can be easily treated through phytoremediation, and the biomass that is formed during the process can be further applied in biodiesel production. Thus, bioenergy crops such as *Brassica* species, which are known to accumulate toxic metals, are increasingly suitable for this purpose. Some plants can accumulate pollutants in tissues [28]. The Jerusalem artichoke (*Helianthus tuberosus* L.) is known to be an energy crop that is used for the phytoremediation of soils contaminated by heavy metals. It is more suitable for the phytoextraction of heavy metals among the energy crops. The highest heavy metal uptake was observed at a dose of 60 Mg DMha^−1^ in the Jerusalem artichoke [29].

Hyperaccumulating plants are fit to grow on soils contaminated with heavy metals and can be used to remove pollutants [19]. Plants that contain greater than 10,000 mg/kg dry weight of Zn or Mn or more than 1000 mg/kg dry weight of Ni, Cu, or Pb or greater than 100 mg/kg dry weight of Cd in contaminated areas are considered as hyperaccumulating plants. There are several families of plants that are known to hyperaccumulate toxic heavy metals. Some such important plant families are *Lamiaceae*, Fabaceae, Scrophulariaceae, Asteraceae, Euphorbiaceae, and Brassicaceae, which are usually used in the phytoremediation processes. Other than that, there have been reports of heavy metal hyperaccumulation in about 500 plant species [30]. Plants with greater hyperaccumulating abilities include *Alyssum bertolonii*, *Thlaspi caerulescens, Calendula officinalis*, and *Tagetes erecta* [31]. Higher concentrations of Ni, Zn, and Cd are best gathered by *Thlaspi caerulescens* [32]. This plant can accumulate 500–52,000 mg kg^−1^ of Zn and 0.3–1020 mg kg^−1^ of Cd. Trees are more suitable for phytoremediation because of their greater root systems and biomass [19], although trees take more time in accumulating metals, even in low quantities. This issue could be solved by using fast-growing plants instead of trees [33]. For an example, the *Poplus alba* is a deciduous tree, which can accumulate zinc (Zn) in different plant parts such as leaves, stems, and roots. An increasing trend of Zn accumulation was observed in the leaves of *Poplus alba* with the application of SDS [34]. Phytoremediation depends on the ability of the plants to gather increased levels of toxic metals within their tissues [35]. Some plants contain enzymes that can break down a number of organic compounds. However, pollutants that are inorganic cannot be degraded with these enzymes. Thus, there is a need to ensure that inorganic pollutants be less available in soils or extracted and accumulated in different parts of the plants and also reduce volatile versions of inorganic pollutants [36]. Energy recovery strategies can be used to produce bioenergy from plant biomass, such as to form biodiesel. Fuel gas, char, and bio-oil can be produced via pyrolysis, during which the biomass undergoes thermal degradation without oxygen [37]. Soils greatly polluted with toxic heavy metals can be remediated easily by growing plants that are tolerant to more than one heavy metal, can produce a good amount of harvestable biomass with enhanced growth rates, and are highly competitive [30].

#### 2.2.2. Problems with Heavy Metals of Remediation with Phytoremediation

Phytoremediation performance could be affected by several processes such as the rate of contaminant uptake by plant roots, the availability of toxic metal ions in the soil, and the root-to-shoot movement of the metal ions [38]. Plants store the heavy metals in the different parts of the plant such as in the leaf, stem, and root [26]. Although phytoremediation is considered as a green technology, it has some problems or limitations in the case of remediation of soils. According to Koptsik [39], there are some problems or limitations of phytoremediation as follows:

- It depends on the local climate, weather, and seasonal conditions.
- It requires more time to remediate pollutants from the soil.
- It is suitable only for low-polluted territories.
- It depends on the depth of the root system and solubility of the pollutants.
- Pollutants may enter the trophic chains and adjacent media.

## 3. Assistance of Chemical Amendments to Increase the Efficiency of Phytoremediation

**Figure 2:**
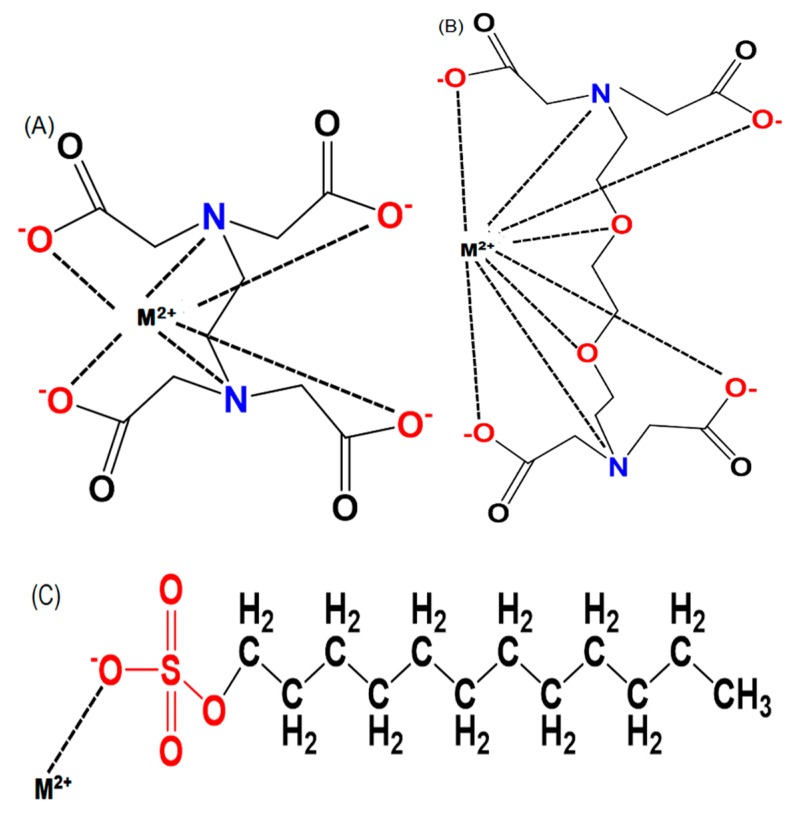
Chemical structure of (**A**) ethylene diamine tetraacetic acid (EDTA), (**B**) ethylene glycol tetraacetic acid (EGTA), (**C**) sodium dodecyl sulfate (SDS), and binding with metals, M^2+^(Cd^2+^, Pb^2+^ etc.).

High heavy metal concentrations in plant tissues and biomass are considered as a key factor for the successful phytoremediation of heavy metal polluted soils [40]. The natural levels of heavy metals are relatively high in the Earth’s crust, and most of these are phytoavailable and low soluble [41]. Chemical amendments have a key role to compensate for relatively low heavy metal availability in soil, and it helps the plants uptake and translocate metals toward the shoot [42]. Different kinds of chemical amendments or chelating agents have been used and tested to increase the bioavailability of metals in plants and facilitate the transport of metals between the roots and shoots. The most important of the three chemical amendments are EDTA, EGTA, and SDS, which have been widely used in recent years (Figure 2).

### 3.1. Assisting of Phytoremediation by EDTA

**Figure 3:**
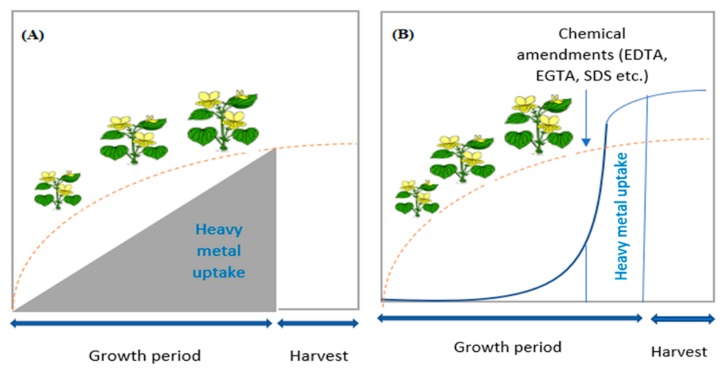
Chemical amendments assisting the heavy metal uptake in the plants, adapted from Souza et al. [43]. Figure (**A**) depicts that a hyperaccumulator plant accumulates the heavy metal gradually and constantly during the entire life cycle of the plant, whereas Figure (**B**) shows that the heavy metal behavior of a non-hyperaccumulator plant is relatively slow, but the metal uptake increases quickly after the application of chemical amendments along with metals.

Ethylenediamine tetraacetic acid, also known as EDTA, is used frequently in the agricultural sector due to its ability to mobilize heavy metals. EDTA enhances metal uptake through the roots and also supports metal xylem loading (Figure 3).

This has also been reported in previous studies [44]. The increased uptake occurs due to the production of soluble metal–EDTA complexes [45].Most plants are able to take up metal–EDTA complexes [46], especially hyperaccumulator species [47]. The effect of EDTA depends on the plant species, heavy metal, and type of soil, ranging from 0 to 200-fold higher accumulations [42]. Cu, Zn, Pb, Ni, and Cd uptake was enhanced by EDTA in *Zea mays* and *Lolium perenne* [48].Lead accumulation increased fourfold in the *Sedum alfredii* roots [49] and twofold in *Vicia faba* seedlings in a 24-h experiment [50].

The metal uptake rate or concentration in plants depends on the chemical amendments dose during the application of chemical amendments. The uptake of toxic metals rate may reach a maximum in plants at a certain amendments dose level. Before treatment, the lead (Pb) concentration was 0.025 mg/g in *Typha* sp. The concentration was increased to 0.846 mg/g, while 1 mL of EDTA was added along with 1 ppm of lead. Similarly, the copper (Cu) was increased 0.030 mg/g to 0.522 mg/g, when 1 mL of EDTA was added in combination with 1 ppm of Cu. Cd concentration was increased by 51.98% in the shoot during the application of EDTA with Cd [45].

In another study, EDTA caused a 15-fold increased uptake of lead in the roots of *Vetiveria zizanioides*. The concentrations of EDTA solution (0–10 mmol∙kg^−1^ soil) were added under the exposure of 1000 mg∙kg^−1^ of Pb in the form of Pb(NO_3_)_2_ for 14 days. They concluded that 10 mmol∙kg^−1^ of EDTA treatment was best regarding the lead (Pb) uptake in *Vetiveria zizanioides* [51]. The maximum Pb concentrations in the shoot were found in *Canavalia ensiformis* L. when 0.5 g∙kg^−1^ of EDTA was used for 40 days with the application of 1800 mg∙kg^−1^ of Pb as Pb(NO_3_)_2_ [52]. A study showed a higher uptake of lead, but no other heavy metals from contaminated soils [53]. Under the exposure of Cd as CdCl_2_ (50 mg∙kg^−1^), the shoot concentrations of Cd significantly increased at a rate of 0.5 g∙kg^−1^ EDTA in *Helianthus annuus* [54] (Table 1).

Phytoextraction using EDTA can be made more efficient with the proper mix of organic chelators, metals, and appropriate plant species [70].

The mechanism of EDTA increasing metal uptake is not fully understood yet. There are various steps involved in the entrance of metals from the soil to the roots, which determines the rate of uptake and also the capability of a plant to take in heavy metals. The uptake of metals into the roots involves: (i) the movement of soluble metals to plant roots through mass flow or diffusion [71], (ii) adsorption on roots, and (iii) attachment to functional groups within the rhizoderm cell surface [72]. The adsorption of metals into the plant root surface has been observed in various studies [73]. The metal–EDTA complexes form affects almost all of the steps previously mentioned of metals uptaking through plant roots. Initially, EDTA allows the diffusion of metals through the roots by (i) increasing their concentration in soil by desorbing metals and (ii) lowering the apparent diffusion coefficient of metals in metal–EDTA complex forms [71]. Since metal–EDTA complexes carry a neutral charge, they are not attached or blocked by polysaccharides or carboxyl groups in the rhizoderm cell surface. In this way, EDTA allows the movement of metals directly into the roots. However, there have been various hypotheses about whether metal–EDTA complexes dissociate just before entering the plant roots or enter as they are [74]. In some studies, it was shown that EDTA form complexes in solution, then enter plants [61]. A study involving 14C-labeling showed that indeed, the full metal–EDTA complex is absorbed, with particular selectivity toward lower charged complexes in Swiss chard. Sarret et al. [75] mentioned that these metal–EDTA complexes are nontoxic and break down after entering the roots, forming free heavy metal ions that could induce phytotoxicity. Inductively coupled plasma mass spectrometry (ICP-MS) analysis of xylem sap showed the presence of metal–EDTA complexes and the absence of EDTA individually in *Hordeum vulgare* grown in contaminated soil amended with EDTA. Schaider et al. [76] showed the presence of complexes in xylem sap such as Cd–EDTA, Pb–EDTA, and Fe–EDTA.

### 3.2. Assisting of Phytoremediation by EGTA

EGTA, or ethylene glycol tetraacetic acid, is a widely used chelating agent. Similar to EDTA, the four carboxyl groups dissociate and produce four protons (Figure 2). The two N atoms of the two amino groups have unshared pairs of electrons each. EGTA has been shown to have more affinity for Ca ions, but less affinity toward Mg ions. Such synthetic chelators have been successfully used for phytoextraction [77]. The factor to consider for using EGTA is that it increases the uptake of heavy metals by plants more efficiently. Sakouhi et al. [66] reported that applying EGTA increased Pb accumulation by more than 80% in parts of *Cicer arietinum* plants above ground. After the application of 1 mmol kg^−1^ of EGTA in *A. rosea,* the maximum total Cd content was observed, which was increased by 72% [68]. In *Mirabilis Jalapa*, Cd concentration was increased by 43.27% in the shoot under EGTA treatment along with Cd (25 mg∙kg^−1^) [64]. The Cd concentration was at a maximum in the shoots when 1.0 mmol∙kg^−1^ was used. In *Calendula officinalis*, 30–100 mg∙kg^−1^ of Cd as CdCl_2_ was added to the soil, and it was observed that the total Cd increases up to 217% with the application of EGTA alone. They concluded that the use of 1.0 mmol kg^−1^ EGTA showed the greatest effect among the treatments [55] (Table 1).

### 3.3. Assisting of Phytoremediation by SDS

SDS is a surfactant that is most commonly used in detergents, but it can also be used for heavy metal and organic contaminant remediation from soil [34]. SDS can ameliorate solubilities of various hydrocarbons and heavy metals such as zinc, cadmium, lead, and copper, making their removal easier, both in phytoremediation trails involving herbaceous species [78] and soil flushing [79]. Surfactants contain a hydrophobic portion that has less affinity for aqueous solutions, and the hydrophilic polar portion, which has a higher affinity for aqueous solutions. Thus, surfactants are amphiphilic. Anionic surfactants such as SDS are amphipathic, as they can interact with both non-polar and polar macromolecules, causing membrane damage and even oxidative stress [80]. However, the direct involvement of SDS in plant remediation trials has not yet been widely investigated, because some strains of *Pseudomonas* can degrade SDS by using it as a carbon source [81,82] and photoelectrochemical reactions [83]. It has been observed that SDS can increase the dry biomass of plants such as *Althaea rosea*, and also promote Cd accumulation in roots and shoots [65]. *Calendula* and *Althaea rosea* provide some evidence that Zn accumulation changes with the presence of SDS [34]. When 1 mM of Zn was added in combination with 0.5 mM of SDS, the Zn translocation was increased toward basal leaves in *Poplus alba* [34]. The maximum Cd concentration was observed in the shoots and roots in *Althaea rosea*, when 1.0 mmol kg^−1^ was added among the single SDS treatments (0.5 mmol∙kg^−1^ to 2 mmol∙kg^−1^) [68]. In *Calendula officinalis*, when the applied soil Cd contamination was 30 mgkg^−1^, the Cd concentrations in the shoot increased significantly under 0.5–2.0 mmolkg^−1^ SDS treatments. For the higher concentration of 100 mg∙kg^−1^ Cd as CdCl_2_, the application of SDS (0.5–2.0 mmol∙kg^−1^) was observed to increase the Cd concentrations in the shoot. They concluded that the maximal shoot Cd concentrations were observed when 2.0 mmol∙kg^−1^ SDS were used [55] (Table 1).

## 4. Challenges with Chemical Amendments

Chemical amendments are useful in phytoremediation methods used to phytoextract heavy metals from contaminated soil; however, adding EDTA, EGTA, and SDS also have a few limitations [84], such as toxic effects toward soil microbes [85], soil enzyme activities, and on cultivated plant species [86]. Due to metal mobilization during extended periods, the chemical-assisted phytoextraction increases the risk of adverse environmental effects. Chemical amendments can disrupt chemical properties and the physical structures of soil by dissolving minerals.

EDTA and its metal complexes are not just highly toxic, but also non-biodegradable and could persist for many weeks [87]. EDTA is an exogenous substance that has adverse environmental effects on soils. EDTA is said to persist within the soil for six months or more [86]. EDTA-facilitated metal movement through soil could occur post-growing season. A monitoring study conducted by [88] mentioned a similar movement of metals such as Zn, Cu, Pb, and Cd using EDTA post-treatment while observing the behavior of heavy metals. Grcman et al. [89] observed that EDTA could leave phytotoxic effects on plants such as red clover. Luo et al. [90] reported similar phytotoxic effects by EDTA even after six months since addition to the soil.

A recent study by Krujatz [91] found that EDTA reduced the toxicity of Ni^2+^ and Cd^2+^ within the stoichiometric ratio, but still inhibited the growth of *Pseudomonas brassicacearum* above the ratio. EDTA also has potentially toxic effects on fungi and bacteria living in soil [88] and plants [84]. Ruley et al. [92] observed lower photosynthetic activity in seedlings of *Sesbania drummondii* that were exposed to EDTA solutions. The toxicity of metals induced by EDTA is due to increased metal uptake [93]. Other phytotoxic effects caused by EDTA may occur due to enhanced metal uptake by plants [94]. Other authors reported results of lower plant biomass when EDTA is present [95]. The toxicity symptom seen in *Brassica juncea* and *Lolium perenne* showed a significant decrease in their biomass [87]. In *Typha angustifolia,* there was a significant decrease in biomass and plant height, resulting in stunted growth [96].

Metal leaching enhanced by chemical amendments depends on various factors, such as:

- Plant-related: root type, species, type and amount of root exudates, age [52,97];
- Soil related: soil texture, pH, organic contents, competing ions, carbonates, biological and microbial conditions, soil water holding capacity, cation exchange capacity (CEC), soil redox potential, soil-buffering capacity [98];
- Metal related: concentration and type of metal, EDTA, EGTA, and SDS-binding capacity to a specific metal, whether single or more than one metal contamination [61,99].

## 5. Conclusions and Future Direction

Heavy metals can cause serious environmental pollution, as they can accumulate in soils and persist for a long time, and even possibly enter the food chain. The chemical amendment-induced remediation of heavy metals by plant species is an effective technique, because it has high binding capacities toward the majority of the heavy metals. Among the three chemical amendments, EDTA is known to be the most efficient organic ligand that can increase metal uptake, solubilization, and translocation, as it can form highly stable and soluble metal–EDTA complexes. Most probably, iron (Fe) is the best metal to bind with EDTA due to its high affinity to ferric ions. Metal phytoremediation enhanced by EDTA, EGTA, and SDS can be affected depending on various biogeochemical processes found in plants, metal, and the soil. Chemical amendment capacity is an important aspect, in order to reduce the time and cost needed for heavy metal contaminated soil. This can be done by increasing the heavy metal bioaccumulation index in plants.

## Figures and Tables

**Table 1:** Effects of heavy metals on the plants with different growing conditions along with chemical amendments.

	Heavy	Growing	Chemical	References
Scientific Name	Metals	Conditions	Amendments	
*Calendula*	Cd	Pot	EDTA, EGTA,	[55]
*officinalis*			SDS	
*Tagetes erecta*	Pb	Pot	EDTA	[56]
*Impatiens*	Cd	Pot	EDTA	[57]
*walleriana*				
*Medicago sativa*	Cr	Pot	EDTA	[58]
*Tribulus terrestris*	Cd, Pb	Pot	EDTA	[59]
*Helianthus annuus*	Cd, Ni	Pot	EDTA	[54]
*Dianthus chinensis*	Cd, Zn, Pb	Pot	EDTA	[60]
*Vetiver zizanioides*	Cd, Zn, Pb	Pot	EDTA	[60]
*Canavalia ensiformis* L.	Pb	Pot	EDTA	[52]
*Brassica carinata*	Cd, Cr, Pb	Pot	EDTA	[44]
*Brassica juncea*	Cd, Cr, Pb	Pot	EDTA	[44]
*Brassica juncea*	Pb	Pot	EDTA	[61]
*Phaseolus vulgaris* L.	Pb, Zn, Cu	Pot	EDTA	[62]
*Zea mays* L. cv.	Pb, Zn, Cu	Pot	EDTA	[62]
Nongda 108				
*Brassica juncea* L.	Pb, Zn	Pot	EDTA	[63]
*Czern.*				
*Triticum aestivum*	Pb, Zn	Pot	EDTA	[63]
*Mirabilis jalapa* L.	Cd	Pot	EDTA, EGTA	[64]
*Althaea rosea*	Cd	Pot	EDTA, EGTA, SDS	[65]
*Mirabilis jalapa*	Cd	Pot	EDTA, EGTA	[64]
*Cicer arietinum*	Cd	Pot	EGTA	[66]
*Sesbania exaltata*	Pb	Pot	EDTA, EGTA	[67]
*Calendula officinalis*	Cd	Pot	EGTA, SDS	[68]
*Althaea rosea*	Cd	Pot	EGTA, SDS	[68]
*Halimione portulacoides*	Cu	Field	SDS	[69]
*Populus alba*	Zn	Pot	SDS	[34]
